# Neonatal and maternal outcomes following midtrimester preterm premature rupture of the membranes: a retrospective cohort study

**DOI:** 10.1186/s12884-016-0813-3

**Published:** 2016-01-29

**Authors:** Laura Aoife Linehan, Jennifer Walsh, Aoife Morris, Louise Kenny, Keelin O’Donoghue, Eugene Dempsey, Noirin Russell

**Affiliations:** The Department of Obstetrics and Gynaecology, University College Cork and Cork University Hospital, Cork, Ireland; The Irish Centre for Fetal and Neonatal Translational Research, Cork, Ireland; The Department of Paediatrics and Child Health, University College Cork, Cork, Ireland

**Keywords:** Preterm birth, Preterm premature rupture of membranes, Sepsis

## Abstract

**Background:**

Preterm premature rupture of membranes (PPROM) complicates 1 % of all pregnancies and occurs in one third of all preterm deliveries. Midtrimester PPROM is often followed by spontaneous miscarriage and elective termination of ongoing pregnancies is offered in many countries. The aim of this retrospective descriptive cohort study was to investigate the natural history of midtrimester PPROM in a jurisdiction where termination of pregnancy in the absence of maternal compromise is unavailable.

**Methods:**

Cases of midtrimester PPROM diagnosed between 14 and 23 + 6 weeks’ gestation during April 2007 to June 2012 were identified following a manual search of all birth registers, pregnancy loss registers, annual reports, ultrasound reports, emergency room registers and neonatal death certificates at Cork University Maternity Hospital - a large (circa 8500 births per annum) tertiary referral maternity hospital in southwest Ireland. Cases where delivery occurred within 24 h of PPROM were excluded.

**Results:**

The prevalence of midtrimester PPROM was 0.1 % (42 cases/44,667 births). The mean gestation at PPROM was 18 weeks. The mean gestation at delivery was 20 + 5 weeks, with an average latency period of 13 days.

Ten infants were born alive (23 %; 10/42). The remainder (77 %; 32/42) died in utero or intrapartum. Nine infants were resuscitated. Two infants survived to discharge. The overall mortality rate was 95 % (40/42).

Five women had clinical chorioamnionitis (12 %; 5/42) but 69 % demonstrated histological chorioamnionitis. One woman developed sepsis (2.4 %; 1/42). Other maternal complications included requirement of intravenous antibiotic treatment (38 %; 17/42), retained placenta (21 %, 9/42) and post-partum haemorrhage (12 %; 5/42).

**Conclusions:**

This study provides useful and contemporary data on midtrimester PPROM. Whilst fetal and neonatal mortality is high, long-term survival is not impossible. The increased risk of maternal morbidity necessitates close surveillance.

**Electronic supplementary material:**

The online version of this article (doi:10.1186/s12884-016-0813-3) contains supplementary material, which is available to authorized users.

## Background

Midtrimester preterm premature rupture of membranes (PPROM) is an uncommon complication, occurring in less than 1 % of pregnancies [1]. PPROM is an important contributor to perinatal mortality and morbidity; in pregnancies that continue following PPROM at early gestations, morbidity is high among surviving neonates with problems including respiratory distress syndrome, pulmonary hypoplasia, intraventricular haemorrhage and limb contractures [2]. Pregnancies complicated by PPROM early in pregnancy, when the risk of pulmonary hypoplasia is highest, present a counselling and management dilemma. It is difficult to predict the eventual outcome as many factors impact on this – the development of sepsis, the eventual gestational age at delivery and the degree of oligohydramnios. There is a wide variety in chorioamnionitis rates and survival rates quoted in the literature. Chorioamnionitis ranged from 28 to 42 % [3, 4], whilst survival rates quoted range from 6.25 [3] to 100 % [5], dependant on gestation. Advances in neonatal care, particularly intensive care to those at the threshold of viability, have dramatically enhanced survival rates. These changes, which reflect a multimodal approach to care, include advances in newborn stabilisation, surfactant administration, optimising respiratory support, the use of nitric oxide and reduction in associated morbidities such as infection and intraventricular haemorrhage and the use of probiotics to reduce necrotising enterocolitis (NEC) [6].

There is a paucity of contemporary evidence about the natural history of these pregnancies as therapeutic termination of pregnancy is routinely offered as standard care in many countries. Termination of pregnancy was not available in Ireland during the time period of this study. However, the publication of the Protection of Life during Pregnancy Act in 2013, provided clarification that termination of pregnancy may be performed if there is a “real and substantial risk of loss of the woman’s life from a physical illness” [7]. There remains an absence of clear guidance if there is no imminent threat to maternal life or health, even in the case of fatal fetal abnormality or where prognosis for the fetus is poor.

Thus, in Ireland when PPROM occurs at a pre-viable gestation but the fetus remains alive, management is problematic. Parents are counselled regarding the guarded prognosis and a care plan is established to provide regular monitoring for the woman. This usually involves regular fetal heart checks via ultrasound and close surveillance for signs and symptoms of maternal sepsis. If there is evidence of developing infection, it may be necessary to induce delivery. This is generally done medically using mifepristone and misoprostol. There is a paucity of information with which to counsel parents regarding fetal and neonatal survival, long term health outcome of surviving infants and the risk of maternal complications, particularly chorioamnionitis and sepsis, in these cases following PPROM at less than 24 weeks’ gestation.

Our objective was to establish the natural history of midtrimester PPROM. We aimed to establish how many women in our hospital experienced midtrimester PPROM and to establish the associated neonatal and maternal health outcomes. We aimed to provide information to assist clinicians to accurately counsel women about maternal and fetal risks associated with midtrimester PPROM.

## Methods

This study was a retrospective descriptive cohort study. Ethical approval was obtained from The Clinical Research and Ethics Committee of the Cork Teaching Hospitals in November 2010, (ref no: ECM 4 (o) 07/12/10), prior to study commencement. The committee deemed that informed written or verbal consent was not required. All women who presented to Cork University Maternity Hospital between April 2007 and June 2012 with midtrimester PPROM were included. Midtrimester was defined as 14 + 0 to 23 + 6 weeks’ gestation. Women who delivered within 24 h of rupture of membranes were not included as we wished to exclude those with preterm birth without prolonged rupture of membranes and those where a PPROM is rapidly followed by a miscarriage. We specifically wished to study those patients who experience a midtrimester prolonged PPROM but do not deliver rapidly so that we could best advise this cohort about the exact risks this poses for them and their baby. A manual search was performed of all birth registers, pregnancy loss registers, annual reports, ultrasound reports, emergency room registers and neonatal death certificates of Cork University Maternity Hospital from April 2007 to June 2012. Cases of midtrimester PPROM were identified and a retrospective chart review was performed. In order to complete data collection on these cases, laboratory and radiology databases were also reviewed.

Our primary outcome measures were gestation at PPROM, gestation at delivery, latency period, neonatal survival rate and neonatal and maternal morbidity. The study was adherent to the STROBE criteria as outlined in Additional file 1. 

## Results

We identified 42 cases of ongoing pregnancy after midtrimester PPROM during the study period, during which 44,667 births occurred. This gives a prevalence of 0.1 %. Maternal demographics are outlined in Table 1. A PPROM was diagnosed when a sterile speculum examination clearly demonstrated liquor to be present in the posterior fornix of the vagina. An ultrasound was also performed to confirm oligohydramnios.

**Table 1:** Maternal Demographics

Maternal age	Average: 32	(Range 19–42, *n* = 42)
Race	Caucasian 31	(*n* = 38)
	African 7	
BMI (kg/m^2^)	Underweight 4	Average BMI 28
	Normal 13	(*n* = 32, range 17–46)
	Overweight 8	
	Obese 5	
	Morbidly Obese 2	
Smoker	Pre-pregnancy 9	
	Pregnancy 4	
Alcohol	Pregnancy 7 (1–10units/wk.)	
Recreational Drug Use	None	

The majority of women had no significant underlying medical conditions (*n* = 35). One woman had antiphospholipid antibodies, one had hypothyroidism, two women had type 2 Diabetes Mellitus with Body Mass Indices (BMI; kg/m^2^) of 40 and 45, one woman had multiple sclerosis, one woman had a PT20210 mutation (an inherited genetic mutation involving the prothrombin gene which increases the risk of thrombosis 2–3 fold) and one woman was HIV positive.

The majority of women (60 %, *n* = 26) had attended for a dating ultrasound scan prior to PPROM. The remainder had their first ultrasound scan at diagnosis of PPROM and dates were assigned via measurement of the bi-parietal diameter, head circumference and femur length. Obstetric history is outlined in Table 2. Of note, 7 % (3/42) had experienced prior PPROM, 9.5 % (4/42) had a prior preterm birth, 4.75 % (2/42) had a previous midtrimester miscarriage and 7 % (3/42) had a previous third trimester loss. Three further women experienced consecutive midtrimester PPROM during the study period and are included twice.

**Table 2:** Obstetric History

Gravidity	Average 2.6 (range 1–7)
Parity	Average 1 (range 0–3)
Primigravidas	12 (29 %)
Prior Preterm Rupture of Membranes	3 (7 %)
	i. 21/40 ii. 27/40 iii. 36/40
Prior Preterm Birth	4 (9.5 %)
	i. 35/40 and 36/40 (IOL for IUD) ii. 28/40 iii. 36/40 iv. 36/40
Intrauterine Death	1 (36/40)
Stillbirth	1 (term)
Neonatal Death	1 (33/40)
Midtrimester Miscarriage	2 (Both at 18/40)
First Trimester Miscarriage	16 (38 %)
• 2 or More	5 (12 %)

All women in our study received oral antibiotics, usually in the form of oral erythromycin. This is in keeping with the Royal College of Obstetricians and Gynaecologists (RCOG) Green Top Guideline No. 44 “Preterm Prelabour Rupture of Membranes” [8] which recommends that erythromycin is given for 10 days following a diagnosis of PPROM. In this scenario, antibiotics were prescribed in the maternal interest due to the risk of sepsis. The majority of women (80 %; 37/42) remained as inpatients to receive this treatment, with 25 women (58 %) delivering in this time period. The average length of stay was 8 nights (range 1–36). Patients received a weekly ultrasound scan to assess fetal wellbeing. The average amniotic fluid index at diagnosis of PPROM was 1 cm (range 0–2, *n* = 12). Antenatal corticosteroids were routinely administered at 24 weeks gestation. Thirty women delivered before 24 weeks and did not receive corticosteroids (range 15 + 6–23 + 3 weeks). Twelve women received intramuscular dexamethasone (28 %) and 83 % of these women (10/12) delivered a live infant. Two of these infants delivered at 23 + 6 weeks. One infant was not actively resuscitated and the other infant lived for 21 days. The single long term surviving infant in this cohort delivered at 23 + 3 weeks after PPROM at 23 + 1 weeks and did not receive antenatal steroids.

One woman presented with symptoms of acute chorioamnionitis and sepsis having experienced PPROM at home 2 days prior to presentation. She was induced in the maternal interest at 20 + 5 weeks and remained an inpatient for intravenous treatment and observation for 3 days, going on to make an uneventful recovery. A placental swab later indicated Group B streptococcal infection. Sixteen other women required intravenous antibiotics but were managed at ward level, without complications.

Twelve percent (*n* = 5) of women suffered clinical chorioamnionitis, but histological chorioamnionitis was found in 69 % of examined placentas. Twenty-five women had recorded treatment with antibiotics. Fourteen had oral erythromycin only, 16 required intravenous antibiotics, namely benzylpenicillin, co-amoxiclav, metronidazole, clindamycin and gentamicin.

Over 20 % of mothers, (9/42), experienced a retained placenta following delivery, thus requiring manual removal. Additionally, 13.9 % (6/42) of women had retained products of conception, four were treated conservatively with antibiotics and two required a surgical uterine evacuation. Other maternal complications are outlined in Table 3. Two women required blood transfusion related to manual removal of placenta.

**Table 3:** Other Maternal Complications

Non-Substantial Ante-Partum Haemorrhage(NSAPH) Prior To PPROM	18 (42 %)
Haemorrhage Following PPROM	18 (42 %)
Suspected Abruption	1 (Emergency Caesarean Delivery At 23 + 3)
Post-Partum Haemorrhage	5 (12 %)
Blood Transfusion	3 (7 %)

The mean gestation at PPROM was 18 weeks (range 15 + 5–23 + 6, *n* = 37). Five women were unsure of exact date of PPROM. The average interval from PPROM to delivery was 13 days (range 1.1–85 days, *n* = 37) and the average gestation at delivery was 20 weeks + 5 days (range 17 + 4–29 + 4, *n* = 42). The average birth weight was 614 g (*n* = 19). Twenty-seven infants had gender recorded, 19 male and eight female. Ten infants were born alive (23 %) with an average birth weight of 740 g (range 440–1100 g) and an average gestational age of 25 + 2 weeks (range 23 + 3–29 + 4). The remainder (76 %; 32/42) died in utero (*n* = 4), intrapartum or at birth due to previability (*n* = 28). No infants weighing less than 500 g were resuscitated. When the infants were grouped according to age, no infants in whom PPROM was recorded to have occurred at less than 17 weeks (*n* = 11) survived, three infants in the 17–21 + 6 group (*n* = 17) were born alive and seven of the infants where PPROM occurred after 22 weeks (*n* = 9) were born alive. Of those born alive, 90 % (9/10) were resuscitated and admitted to the neonatal intensive care unit (NICU). Seven received surfactant. Six required intravenous antibiotics. The average length of stay in NICU was 34.6 days (range 2 h–146 days).

Three infants died within 2 h of birth, including the infant not admitted. One of these infants had been a PPROM at 17 weeks gestation and delivered at 25 + 3 with pulmonary hypoplasia and limb contractures. The other two infants had low APGAR scores at delivery and were treated palliatively without active resuscitation. Complications affecting neonatal survivors are outlined in Table 4.

**Table 4:** Neonatal Complications

Respiratory Distress Syndrome	7 (70 %, 100 % of those surviving beyond 2 h)
Sepsis	3 (30 %)
Coagulase Negative Staphylococcal (CONS) Sepsis	2 (20 %)
Patent Ductus Arteriosus (PDA)	4 (40 %)
Necrotising Enterocolitis (NEC)	2 (20 %)
Intraventricular Haemorrhage(IVH)	3 (30 %)

Seven infants survived at least 7 days. Two infants survived to discharge. Both were female infants. One female infant was born at 29 + 4 after a latency period of 85 days weighing 930 g. She was transferred to another paediatric surgical institution at 20 days of age but subsequently died of Necrotising Enterocolitis at 8 weeks of age. The surviving neonate was born to a 24 year old African woman who was HIV positive. Rupture of membranes occurred at 23 + 1 weeks and delivery was 2 days later at 23 + 3 weeks. The mother developed oligohydramnios and clinical chorioamnionitis. Treatment of this woman involved oral erythromycin, oral co-amoxiclav, intravenous gentamicin, intravenous metronidazole and intravenous ceftriaxone. A female infant weighing 580 g was delivered. Length of stay in the neonatal intensive care unit was 146 days. Complications following birth included respiratory distress syndrome, patent ductus arteriosus, patent foramen ovale, grade two intraventricular haemorrhage and multiple seizures. The infant was discharged home alive with a diagnosis of chronic lung disease but is currently a healthy 4 year old who had met all her developmental milestones on discharge from neonatology follow-up.

After delivery, women were investigated for potential underlying causes of second trimester loss and also screened for infection. These investigations included blood counts, high vaginal swabs, mid-stream urinalysis, placental swabs, serology for cytomegalovirus, toxoplasma, syphilis, rubella and parvovirus B19, thyroid function tests, thrombophilia and autoantibody screens. Cytogenetics, post mortem and placental histological examination were also offered. All women were followed up postnatally in the pregnancy loss clinic to discuss their pregnancy events and outcome, to follow up on investigations and to formulate a management plan for subsequent pregnancies. They were also supported by the specialist midwifes in bereavement and loss and offered formal counselling support where desired.

Placental histology was available in 32 cases. Although only five women had clinical symptoms of chorioamnionitis (12 %; 5/42) there was histological chorioamnionitis in 69 % of examined placentas (22/42). Two further placentas demonstrated retroplacental haemorrhage and one demonstrated haemorrhagic infarction. The remainder were reported as normal (17 %; 7/42). Nine infants had cytogenetic analysis which all demonstrated normal karyotypes. Three infants underwent post mortem examination. These demonstrated anatomically normal infants.

Twenty-eight women (67 %) had high vaginal swabs- two swabs cultured group B streptococcus. Twenty women (48 %) had mid-stream urine samples sent, none of which demonstrated significant infection. Seven women (17 %) had placental swabs taken – one cultured group B streptococcus. Only the woman who developed sepsis had blood cultures taken.

## Discussion

The incidence of midtrimester PPROM was 1 in 1000 pregnancies. The mean gestational age at presentation was 18 weeks and the mean interval to delivery was 13 days. The average age at delivery in our study was 20 + 5, which is remote from viability. The average birth weight was 614 g. Thus survival in this cohort was very unlikely. Thirty-two infants (76 %) died in utero (4/42) or peripartum due to previability (28/42). Ten infants were born alive (23 %; 10/42) with an average birth weight of 740 g (range 440–1100 g) and an average gestational age of 25 + 2 weeks (range 23 + 3–29 + 4). Ninety percent (9/10) of these were actively managed and taken to the NICU. Six of these infants died in the NICU of sepsis, IVH or RDS, hence the overall survival to discharge was 4.76 % (2/42). No infants in whom PPROM was recorded to have occurred at less than 17 weeks (*n* = 11) survived, three infants in the 17–21 + 6 group (17 %, *n* = 17) were born alive and seven of the infants where PPROM occurred after 22 weeks (78 %, *n* = 9) were born alive. Maternal morbidity was low, despite the prolonged latency period and the lack of availability of termination or delivery on maternal request. The incidence of clinical chorioamnionitis was low (14 %; 6/42) but high histologically suggesting a high index of suspicion is required with these women. Whilst 17 women required antibiotics, only one woman developed sepsis. This suggests that it is reasonable to offer women conservative management of PPROM, once they are carefully monitored for signs of sepsis. Although neonatal survival rates are low, women may choose this as a more acceptable alternative to termination of pregnancy.

This study was distinctive in examining PPROM at early gestations. There are few studies examining the outcomes of these pregnancies. Additionally, we provide a unique perspective as elective termination of pregnancy is not routinely offered in Ireland for this indication in the absence of maternal compromise. This study provides significant information on the natural history of PPROM and vital information with which to counsel future affected women.

This is a small retrospective study that refers to a single institutional experience, thus our study is limited by the retrospective nature of data retrieval and the small number of cases available. Furthermore, a proportion of women in our study (5/42) were unsure as to the exact date of PPROM. Thus we must consider unrecognised PPROM may present as preterm labour or late miscarriage and may have been missed in our search. Similarly, late presentations close to viability may have been missed as duration of PPROM was not always well documented in this cohort. These groups may have yielded survivors.

Other studies have included women experiencing PPROM at much later gestations up to 34 weeks [9]. Indeed, this often accounts for their larger sample size (range 16–236) [3, 10]. Similarly as the infants in our study were born at earlier gestations, their survival rate was also lower than other studies. A study by Manuck et al. also examined women experiencing PPROM between 14 and 23 + 6 weeks [11]. Their study was conducted over 6 years however with a larger cohort of 159 patients, presenting at a later mean gestational age (21 + 3) and delivering at a later mean gestation of 24 + 5 and included those delivering 12 h after PPROM. Indeed shorter latency periods and later gestational age may have contributed to the increased survival rate of 56 %. Holmgren et al. found a survival rate of 86 %, but included women who experienced PPROM between 14 + 0 and 32 + 0 weeks [12]. There were lower survival rates in all studies in the earlier PPROM cases but there was a wide variance in figures. Verma et al. had a survival rate of 18.3 % but examined pregnancies where PPROM occurred between 18 and 23 weeks only [13]. Conversely, Loeb et al. included pregnancies complicated by PPROM between 20 and 24 weeks gestation and found an overall survival rate of 6.25 % [3]. Survival was 40, 92 and 100 % of those experiencing PPROM at 14–19, 20–25 and 26–28 weeks respectively by Farooqi et al. [5]. Likewise, Newman et al. found a mortality of 98.8 % in PPROM occurring at 23–24 weeks which fell to 36.6 % in the 25–27week group [10]. Xiao reported a similarly high mortality rate of 82 % in their PPROM cases at less than 22 weeks gestation (14–21.9 weeks) [14]. Margato et al. also found that PPROM occurring at gestations less than 20 weeks had a lower survival rate [15]. This is consistent with our own findings which showed a 100 % mortality rate in those with PPROM at less than 17 weeks, but 78 % of infants experiencing PPROM after 22 weeks were born alive. Latency periods also varied widely (1.25 – 105 days) [2]. The consensus was that longer latency periods had a positive impact on survival, allowing time for corticosteroid administration and antibiotics, but equally increased the risk of chorioamnionitis [5, 13].

Chorioamnionitis rates in our study were lower than others at 13 %. Rates in other studies ranged from 28 to 42 % [4, 5]. Similarly, Loeb described a much higher incidence of histological versus clinical chorioamnionitis (85 % vs 39 %) [3]. There was a 20 % incidence of retained placenta. This is much higher than Verma et al. who reported an incidence of retained placenta of 9.09 % [13]. Of the women with retained placenta, four had histological chorioamnionitis (44 %, 4/9) and one had clinical signs of chorioamnionitis (11 %, 1/9). The remaining women (*n* = 4) did not have placental histology available. There appeared to be no association between latency period and retained placenta. With the exception of one woman with a latency period of 85 days, the remainder had an average latency of 4.2 days (*n* = 5, *r* = 2–7).

Neonatal complications were comparable to other studies. All infants transferred to the NICU had significant respiratory morbidity and were managed with mechanical ventilation, nitric oxide and surfactant. Verma et al. reported a similar finding with an incidence of RDS of 100 % [13]. Dinsmoor et al. reported sepsis among 34 % of subjects consistent with our rate of 30 % [1]. IVH occurred in 30 % of our cohort, two of which were grade 4. Other studies reported lower rates of severe IVH [1, 4, 13]. Most studies conceded that neonatal morbidity was high in this cohort and was often serious, with at least one major morbidity present in the range of 37 to 100 % [1, 2, 4, 11–14] of those infants surviving to discharge. Our single survivor had four major complications of prematurity and continues to have respiratory complications, emphasising the importance of antenatal steroids near viability. This places our survival rate of infants born alive at just 10 %, similar to Loeb but lower than other authors who found survival rates of 67–81 % [3, 12].

## Conclusion

In summary our study shows that PPROM prior to viability is a rare complication of pregnancy, but one that carries significant fetal and maternal risks. By focusing on these women of earlier gestation we provide useful data for pregnant women and clinicians regarding these challenging cases. It is clear however that further work needs to be done in this area, involving larger numbers and a collaboration of experience from multiple institutions. There was an inconsistency of investigations, particularly in screening for infection on initial presentation. The introduction of a new national guideline on PPROM [16] and the Irish Maternity Early Warning System (I-MEWS) [17] should help to improve initial management as well as detection and prevention of sepsis. Future studies should focus on the impact of these measures.

Whilst our overall survival rate was 5 %, 78 % of foetuses in those pregnancies where the PPROM occurred after 22 weeks were born alive. Counselling must be frank regarding the poor prognosis of those remote from viability, but awareness of increasing fetal potential with enduring latency periods should encourage the clinician caring for this challenging cohort. Additionally, advances in neonatal care and therapies such as amnioinfusion or amniopatch may offer increased hope of survival in the future [18]. Acknowledging the risk to mother and fetus from infection should instigate due diligence in seeking signs and symptoms of sepsis. Recent changes to Irish legislation add another dimension to counselling and decisions surrounding delivery in the maternal interest in cases of acute chorioamnionitis [7, 16].

## Additional file

Additional file 1:
**Strobe checklist**. (DOC 80 kb)

## Abbreviations

PPROM: preterm premature rupture of membranes
RCOG: royal college of obstetricians and gynaecologists
NICU: neonatal intensive care unit
IVH: intraventricular haemorrhage
RDS: respiratory distress syndrome
NEC: necrotising enterocolitis
NSAPH: non-substantial antepartum haemorrhage
I-MEWS: Irish maternity early warning system
