# Supplementary material for: Neonatal and maternal outcomes following midtrimester preterm premature rupture of the membranes: a retrospective cohort study
Source: BMC Pregnancy Childbirth. 2016 Jan 29;16:25. doi: 10.1186/s12884-016-0813-3 (PMC4734873; doi:10.1186/s12884-016-0813-3)
Supplement: Additional file 1: — Strobe checklist. (DOC 80 kb) [file 12884_2016_813_MOESM1_ESM.doc]

|  | Item No | Recommendation | Please insert check where included or N/A where not applicable |
| --- | --- | --- | --- |
| **Title and abstract** | 1 | (*a*) Indicate the study’s design with a commonly used term in the title or the abstract | ✓ |
|  |  | (*b*) Provide in the abstract an informative and balanced summary of what was done and what was found | ✓ |
| Introduction | | |  |
| Background/rationale | 2 | Explain the scientific background and rationale for the investigation being reported | ✓ |
| Objectives | 3 | State specific objectives, including any prespecified hypotheses | ✓ |
| Methods | | |  |
| Study design | 4 | Present key elements of study design early in the paper | ✓ |
| Setting | 5 | Describe the setting, locations, and relevant dates, including periods of recruitment, treatment, follow-up, and data collection | ✓ |
| Participants | 6 | (*a*) *Cohort study*—Give the eligibility criteria, and the sources and methods of selection of participants. Describe methods of follow-up  *Case-control study*—Give the eligibility criteria, and the sources and methods of case ascertainment and control selection. Give the rationale for the choice of cases and controls  *Cross-sectional study*—Give the eligibility criteria, and the sources and methods of selection of participants | ✓ |
|  |  | (*b*) *Cohort study*—For matched studies, give matching criteria and number of treated and untreated  *Case-control study*—For matched studies, give matching criteria and the number of controls per case   \| Participants \| 13* \| (a) Report numbers of individuals at each stage of study—eg numbers potentially eligible, examined for eligibility, confirmed eligible, included in the study, completing follow-up, and analysed \|  \| \| --- \| --- \| --- \| --- \| \|  \|  \| (b) Give reasons for non-participation at each stage \|  \| \| Descriptive data \| 14* \| (a) Give characteristics of study participants (eg demographic, clinical, social) and information on other treatments and potential confounders \|  \| \|  \|  \| (b) Indicate number of participants with missing data for each variable of interest \|  \| \|  \|  \| (c) *Cohort study*—Summarise follow-up time (eg, average and total amount) \|  \| \| Variables \| 7 \| Clearly define all outcomes, treatments, predictors, potential confounders, and effect modifiers. Give diagnostic criteria, if applicable \|  \| \| Data sources/ measurement \| 8* \| For each variable of interest, give sources of data and details of methods of assessment (measurement). Describe comparability of assessment methods if there is more than one group \|  \| \| Bias \| 9 \| Describe any efforts to address potential sources of bias \|  \| \| Study size \| 10 \| Explain how the study size was arrived at \|  \| \| Quantitative variables \| 11 \| Explain how quantitative variables were handled in the analyses. If applicable, describe which groupings were chosen and why \|  \| \| Statistical methods \| 12 \| (*a*) Describe all statistical methods, including those used to control for confounding \|  \| \| (*b*) Describe any methods used to examine subgroups and interactions \|  \| \| (*c*) Explain how missing data were addressed \|  \| \| (*d*) *Cohort study*—If applicable, explain how loss to follow-up was addressed  *Case-control study*—If applicable, explain how matching of cases and controls was addressed  *Cross-sectional study*—If applicable, describe  analytical methods taking account of sampling strategy \|  \| \| (*e*) Describe any sensitivity analyses \|  \| | n/a  ✓  ✓  ✓  ✓  ✓  ✓  ✓  ✓  ✓  ✓  ✓  n/a  n/a |
